# Acute flaccid myelitis in Europe between 2016 and 2023: indicating the need for better registration

**DOI:** 10.2807/1560-7917.ES.2025.30.21.2400579

**Published:** 2025-05-29

**Authors:** Jelte Helfferich, Cristina Calvo, Ekkehardt Alpeter, Cristina Andrés, Andrés Antón, Melodie Aubart, Stefania Maria Bova, Maria Cabrerizo, Karin von Eije, Stollar Fabiola, Ana Felipe, Ralitsa Iordanova, Marianne Kragh Thomsen, Per Kristian Knudsen, Freek van Loenen, Noemi Lopez, Audrey Mirand, Richard Molenkamp, Sofie Midgley, Raquel Neves, Lubomira Nikolaeva Glomb, Joakim Øverbø, Gülten Öztürk, Paula Palminha, Helle Cecilie Viekilde Pfeiffer, Birgit Prochazka, Carlos Ribeiro, Martine Rodesch, Isabelle Schuffenecker, Jay Shetty, Sandy Siegert, Silje Lae Solberg, Artur Sulik, Dilşad Türkdoğan, Olcay Ünver, Jaco Verweij, Jorgina Vila, Tytti Vuorinen, Ronny Wickström, Thea K Fischer, Heli Harvala, Kimberley S.M. Benschop

**Affiliations:** 1 Department of Neurology, University Medical Center Groningen, University of Groningen, Groningen, The Netherlands; 2 Centro de Investigación Biomédica en red de Enfermedades Infecciosas CIBERINFEC, Instituto Carlos III, Madrid, Spain; 3 Pediatric Infectious and Tropical Diseases Department, La Paz University Hospital and Translational Research Network in Pediatric Infectious Diseases (RITIP), Institute for Health Research IdiPAZ, Autonomous University of Madrid, Madrid, Spain; 4 Federal Department of Home Affairs FDHA, Federal Office of Public Health FOPH, Division of Communicable Disease, Bern, Switzerland; 5 Respiratory Viruses Unit, Virology Section, Microbiology Department, Vall d’Hebron Hospital Universitari, Vall d’Hebron Institut of Research (VHIR), Vall d’Hebron Barcelona Hospital Campus, Barcelona, Spain; 6 Pediatric Neurology Department, Necker-Enfants malades Hospital, AP-HP, Paris, France; 7 Human Genetics of Infectious Diseases, University of Paris Cité, Institut Imagine, Paris, France; 8 Child Neurology Unit, Buzzi Children hospital, Milan, Italy; 9 Polio/Enterovirus National Lab, National Centre of Microbiology, Instituto de Salud Carlos III, Madrid, Spain. CIBERESP and RITIP (Idipaz); 10 Department of Viroscience, Erasmus University Medical Center, Rotterdam, The Netherlands; 11 Division of General Pediatrics, Department of Pediatrics, Gynecology & Obstetrics, University Hospitals of Geneva, Geneva, Switzerland; 12 Member of the SPSU (Swiss paediatric Surveillance Unit) committee, Switzerland; 13 Paediatric Neurology Research Group, Vall d'Hebron Institut de Recerca, Barcelona, Catalonia, Spain; 14 Paediatric Neurology Section, Children's Hospital, Vall d'Hebron Barcelona Hospital Campus, Barcelona, Catalonia, Spain; 15 Department of Pediatrics UMHAT “St. George” - Plovdiv, Plovdiv, Bulgaria; 16 Department of Pediatrics, Medical University of Plovdiv, Plovdiv, Bulgaria; 17 Department of Clinical Microbiology, Aarhus University Hospital, Aarhus, Denmark; 18 Department of Paediatric and Adolescent Medicine and Department of Paediatric Research, Oslo University Hospital, Oslo, Norway; 19 Centro Nacional de Epidemiología, Instituto de Salud Carlos III, Madrid, Spain; 20 CIBERESP, Dpto. Medicina Preventiva y Salud Pública, Universidad Autónoma de Madrid, Madrid, Spain; 21 CHU Clermont-Ferrand, Centre National de référence des entéroviurs et parechovirus-laboratoire coordonnateur, Clermont-Ferrand, France; 22 The Danish WHO National Reference Laboratory for Poliovirus, Section for Virus Genomics, Department of Virus & Microbiological Preparedness, Division of Infectious Disease Diagnostic Preparedness, Copenhagen, Denmark; 23 National Reference Laboratory for Vaccine-Preventable Diseases, Department of Infectious Diseases, National Institute of Health, Doutor Ricardo Jorge, Lisbon, Portugal; 24 National Reference Laboratory for Enteroviruses, National Center for Infectious and Parasitic Diseases, Sofia, Bulgaria; 25 The Norwegian Institute of Public Health (NIPH), Oslo, Norway; 26 Department of pediatric neurology, Marmara University, Istanbul, Türkiye; 27 Department of Pediatrics, Copenhagen University Hospital Hvidovre, Copenhagen, Denmark; 28 Department of Clinical Medicine, University of Copenhagen, Copenhagen, Denmark; 29 AGES, Wien, Austria; 30 Department of pediatrics, Hôpital Erasme, Brussels, Belgium; 31 Department of Virology, National Reference Centre for Enteroviruses and Parechoviruses, Associated Laboratory, Université Claude Bernard Lyon 1, CHU Lyon, Lyon, France; 32 Department of Paediatric Neurosciences, Royal Hospital for Children and Young People, Edinburgh, United Kingdom; 33 Division of Pediatric Pulmonology, Allergology and Endocrinology, Department of Pediatrics and Adolescent Medicine, Medical University of Vienna, Vienna, Austria; 34 Department of Infection Control and Vaccines, Norwegian Institute of Public Health, Oslo, Norway; 35 Department of Pediatric Infectious Diseases, Medical University of Bialystok, Bialystok, Poland; 36 Microvida Laboratory for Medical Microbiology and Immunology, ElisabethTweesteden Hospital, Tilburg, The Netherlands; 37 Infection and Immunity Research Group, Vall d'Hebron Institut de Recerca, Barcelona, Catalonia, Spain; 38 Paediatric Hospitalization Unit, Children's Hospital, Vall d'Hebron Barcelona Hospital Campus, Barcelona, Catalonia, Spain; 39 Department of Clinical Microbiology, Turku University Hospital, Turku, Finland; 40 Institute of Biomedicine, University of Turku, Turku, Finland; 41 Neuropediatric Unit, Department of Women’s and Children’s Health, Karolinska Institutet and Karolinska University Hospital, Stockholm, Sweden; 42 Department of Public Health, Section of Global Health, University of Copenhagen, Copenhagen, Denmark; 43 Department of Clinical Research, North Zealand University Hospital, Hillerød, Denmark; 44 Department of Infection and Immunity, University College of London, London, United Kingdom; 45 Microbiology Services, National Health Service (NHS) Blood and Transplant, London, United Kingdom; 46 Department of Clinical Microbiology, Turku University Hospital, Turku, Finland; 47 Institute of Biomedicine, University of Turku, Turku, Finland; 48 Centre for Infectious Disease Control (CIb), National Institute for Public Health and the Environment (RIVM), Bilthoven, the Netherlands

**Keywords:** Acute flaccid myelitis, Acute flaccid paralysis, Enterovirus D68, Enterovirus A71, Surveillance, Children

## Abstract

**Background:**

Acute flaccid myelitis (AFM) is a rare polio-like condition affecting mainly children and characterised by severe, often persistent, weakness. It is one of several causes of acute flaccid paralysis (AFP), which manifests as acute onset of limb weakness and reduced muscle tone. Some non-polio enteroviruses (EV), such as EV-D68 may cause AFM. Little is known about AFM incidence in Europe.

**Aim:**

We aimed to better understand AFM incidence, aetiology and current surveillance policies in Europe.

**Methods:**

In 28 countries, members of the European non-polio enterovirus network (ENPEN) and a newly established AFM network of clinicians under ENPEN received a survey asking them how AFM surveillance was performed in their countries in 2016−2023 and the numbers of AFM cases including those diagnosed with EV-D68 infection during this period.

**Results:**

Surveillance information was obtained for 16 countries. In eight countries, AFP surveillance initiated for poliomyelitis eradication was still ongoing, while non-polio AFM cases were only systematically reported in Norway. The survey revealed 130 AFM cases for 14 countries, with 48 (37%) EV-D68-laboratory-confirmed. Among the AFM cases, 70% (n = 91) occurred in 2016, 2018 and 2022, when EV-D68 circulation increased.

**Conclusions:**

This report provides some indication of AFM case numbers in Europe since 2016. However, as 15 of 16 countries with AFM monitoring information lacked structural AFM surveillance, numbers should be interpreted with caution. Knowing AFM incidence matters to determine its impact and detect future outbreaks. Thus, the newly established clinical network will develop a European AFM repository.

**Key public health message**

**What did you want to address in this study?**

Acute flaccid myelitis (AFM) is a rare but serious condition involving progressive and often severe limb weakness. Several viruses can cause AFM, and AFM cases have been observed to occur when enterovirus D68 (EV-D68) circulates. Little is known about AFM incidence in Europe, so we wanted to shed light on AFM surveillance policies in different countries there, and on AFM case numbers recorded between 2016 and 2023, as well as their aetiology.

**What have we learnt from this study?**

A survey of members of the European non-polio enterovirus network (ENPEN), which includes a newly established clinical network was conducted in 28 countries. Through the survey we obtained surveillance information from 16 countries. Among these, only Norway had a valid structured surveillance for AFM. The survey revealed 130 AFM cases for 14 countries, with 91 cases (70%) in years 2016, 2018 and 2022, when EV-D68 circulation increased.

**What are the implications of your findings for public health?**

Due to the lack of structured surveillance, the numbers of AFM cases reported through our study should be interpreted with caution. Improved knowledge of the incidence of AFM in European countries is crucial to determine its impact on people’s health, as well as to detect future AFM outbreaks. To this end, the newly established clinical network under ENPEN will develop a European AFM repository, aiming to involve of as many countries as possible.

## Introduction

Acute flaccid myelitis (AFM) is a rare but serious condition impacting the spinal cord, and particularly the grey matter. It is one of several causes of acute flaccid paralysis (AFP), a syndrome characterised by acute onset of limb weakness and reduced muscle tone. Children under 10 years old constitute the main demographic group where AFM occurs [1], and the condition leads to severe deficits that often persist even several years after disease onset. Facial, truncal and respiratory muscles are commonly affected, resulting in mechanical-ventilation dependency in 20 to 40% of cases in the acute phase [2-4]. Weakness is presumably due to damage of anterior horn cells in the spinal cord, because of viral invasion and inflammation [5,6].

Different viruses may cause AFM and, in the last decade, non-polio enteroviruses (EV) D68 and A71 have been associated with the condition. This is based on their frequent identification in patients with AFM, as well as on correlations found between increased detections of these viruses and upsurges of AFM case numbers in populations [2,4,7]. In addition to non-polio-EVs, polioviruses, in particular vaccine-derived strains, are still circulating in the world including in high income countries and may also cause AFM [8].

In Europe, the largest case series of AFM reported in the past 10 years were in 2016 and 2018. In 2016, 29 cases of AFM associated to EV-D68 were observed across 12 European countries [4]. In 2018, 40 cases of AFP were documented in the United Kingdom, nine of whom fulfilled the diagnostic criteria for AFM [9]. Additionally, 34 AFM cases from Türkiye were recorded, with six diagnosed in 2016, one in 2017 and 27 in 2018 [10]. After 2018, only limited numbers of cases of AFM have been described in Europe. While the case series may suggest that several outbreaks or upsurges in AFM incidence could have happened over several years, the exact number of children affected by AFM in Europe is currently largely unknown.

According to a study published in 2016, which focused on the European Union/European Economic Area (EU/EEA), there is no systematic clinical AFM surveillance in most EU/EEA countries. As AFM is one cause of AFP, AFP surveillance may be able to detect AFM cases when further differentiation of the cause of the identified cases is performed. However, AFP surveillance, which was established for polio surveillance is no longer effective in the majority of EU/EEA countries, even though poliovirus infection is notifiable in all of them [11]. Moreover, in its current form, the focus is on exclusion of poliovirus, without further testing or differentiation of the cause.

Concerning laboratory-based non-polio EV surveillance, strategies in Europe are heterogeneous [11]. Through EV surveillance, outbreaks of viruses associated with AFM such as EV-D68 or EV-A71 may be uncovered, but this requires respiratory and faecal specimen collection, as well as testing samples for these viruses and typing. Additionally, to monitor AFM, clinical data from laboratory-confirmed EV-positive specimens, which are often limited, should be used to investigate if the clinical picture is compatible with this condition. As the number of AFM cases reported as part of different EV-D68 and EV-A71 outbreaks is sparse, an EV surveillance approach cannot by itself be used for AFM surveillance [12], however, it may work synergistically with AFM clinical surveillance.

Considering the effect of AFM on individual patients and the possibility of future outbreaks, it is important, in our opinion, to monitor the incidence of AFM and its potential causes [13]. In this study, which covered a period from 2016 to 2023, we aimed to update knowledge on the current surveillance strategies used in Europe for paralytic cases, as well to gain insight into the numbers of AFM cases, including those laboratory-confirmed as EV-D68.

## Methods

Members of the European non-polio enterovirus network (ENPEN) and of the recently established European AFM network under ENPEN, who include clinicians and virologists/microbiologists [14] were sent a brief survey. The survey aimed to gather information about their country’s AFM surveillance strategy, including any changes made following the 2016 upsurge in cases. Furthermore, they were asked to describe in which hospital/institute virological testing was conducted in their country or institute for virus detection and characterisation. The questions in the survey were: (i) In which way is AFM surveillance performed in your country? Has this changed since 2016? (ii) If applicable, in which hospital or institute is the virological testing for your centre performed?

In addition, an inquiry was done for the number of clinically diagnosed AFM cases, identified each year between 2016 and 2023, and the number of cases in which EV-D68 was laboratory-confirmed. Respondents were requested to fill in a table with numbers of cases per year and those not spontaneously specifying whether data were nationally or regionally obtained were subsequently asked to provide this information. 

While the number of AFP cases was not requested in the survey, this information was spontaneously provided by respondents from four countries.

## Results

The survey was sent out to 33 clinicians (paediatricians, neurologists, infectiologists) from 24 institutions and 141 virologists/microbiologists (clinical, molecular, public health) from 79 institutions in a total of 28 countries in Europe. A response was obtained from 22 institutions from 16 countries (Supplementary Figure), of which 18 institutions from 15 respective countries were able to provide data on the number of AFM cases. A map with the countries from which a response was received is shown in the Supplementary Figure.

Information on the number of AFP cases was spontaneously provided by four countries, with for one of these no accompanying AFM case numbers (Switzerland) and for the remaining three, additional AFM case number data (Norway, Spain and Poland (only 2022 and 2023)). In total, 751 cases of AFP were reported. Information about virological testing was not requested nor obtained for these cases.

From 16 countries information on surveillance methods was obtained, with respondents of nine countries involved in national polio surveillance. Surveillance of AFP, initiated for the eradication of poliomyelitis was still active in eight (Austria, Belgium, Italy, Norway, Poland, Spain, Switzerland, Turkey) of the 16 countries, while systematic reporting of non-polio AFM cases is only performed in Norway. Also in Norway, respiratory specimens are obtained from AFP cases since 2014, while for other countries this information was not reported, as it was not asked in the survey. None of the respondents were aware of whether a change in clinical surveillance for AFP/AFM had occurred after the upsurge of AFM cases in 2016. While information on EV surveillance was not specifically included in the questionnaire, respondents from four countries (Austria, Bulgaria, France, Netherlands) voluntarily mentioned that some form of laboratory surveillance for EV was active within their country. Virological testing in suspected AFM cases was either done in local laboratories (12/16) or national reference centres (4/16).

A total of 130 AFM cases were reported by 15 countries. Respondents from eight of these 15 countries were involved in national poliovirus surveillance and respondents from seven countries were providing information based on regional or national networks (n = 5) or personal awareness (n = 2). Among the 130 AFM cases, 70% occurred in the years 2016 (n = 37), 2018 (n = 39) and 2022 (n = 15) (Table, Figure), and 37% (n = 48) were reported to be laboratory-confirmed as EV-D68. In the Table, an overview of the total number of cases per year is provided.

**Table:** Acute flaccid myelitis cases reported through the current study, Europe, 2016–2023 (n = 130 cases)

Country	Profession of respondents	National data	2016		2017		2018		2019		2020		2021		2022		2023		Total AFM cases
			Number of cases of																
			AFM	EV-D68	AFM	EV-D68	AFM	EV-D68	AFM	EV-D68	AFM	EV-D68	AFM	EV-D68	AFM	EV-D68	AFM	EV-D68	
Austria	Clinician virologist^a^	No	NA	NA	NA	NA	NA	NA	NA	NA	NA	NA	NA	NA	1	0	NA	NA	1
Belgium	Clinician	No	0	0	0	0	0	0	1	1	0	0	0	0	0	0	NA	NA	1
Bulgaria	Clinician virologist	No	0	NA	0	NA	0	NA	0	NA	0	NA	0	NA	0	NA	0	NA	0
Denmark	Virologist^a^	NA	NA	NA	NA	NA	1	NA	2	1	NA	NA	NA	NA	NA	NA	2	NA	5
Italy	Clinician	No, regional	4	0	NA	NA	NA	NA	NA	NA	NA	NA	NA	NA	1	1	NA	NA	5
Finland	Virologist^a^	NA	NA	NA	NA	NA	NA	0	NA	NA	NA	NA	NA	NA	0	NA	NA	NA	0
France	Clinician virologist^a^	Yes	4	4	0	0	3	3	2	2	0	0	1	1	2	2	0	0	12
Netherlands	Clinician virologist^a^	Yes	4	2	0	0	3	1	3	2	1	1	4	2	1	1	1	0	17
Norway	Clinician virologist^a^	Yes	3	3	0	0	1	1	0	0	0	0	0	0	0	0	0	0	4
Poland	Clinician	No	NA	NA	NA	NA	NA	NA	NA	NA	NA	NA	NA	NA	4	0	3	0	7
Portugal	Virologist^a^	NA	2	0	2	0	0	0	1	0	0	0	1	0	1	0	0	0	7
Scotland (UK)	Clinician	Yes	5	5	0	0	0	0	0	0	0	0	0	0	1	1	0	0	6
Spain	Clinician virologist^a^	Yes	9	1	2	0	4	1	3	0	1	0	0	0	2	1	3	0	24
Sweden	Clinician	No, regional	0	0	0	0	0	0	1	1	1	1	0	0	2	2	1	1	5
Switzerland	Clinician virologist^a^	NA	NA	NA	NA	NA	NA	NA	NA	NA	NA	NA	NA	NA	NA	NA	NA	NA	NA
Türkiye	Clinician	Yes	6	NA	1	NA	27	6	NA	NA	NA	NA	NA	NA	NA	NA	2	NA	36
**Total**			**37**	**15**	**5**	**0**	**39**	**12**	**13**	**7**	**3**	**2**	**6**	**3**	**15**	**8**	**12**	**1**	**130**

AFM: acute flaccid myelitis; EV-D68: enterovirus D68; NA: not available; UK: United Kingdom.

^a^ Respondents involved in national polio surveillance systems.

The Table shows the number of reported cases of AFM and the number of EV-D68 positive AFM cases, as provided by the different respondents in Europe.

**Figure:**
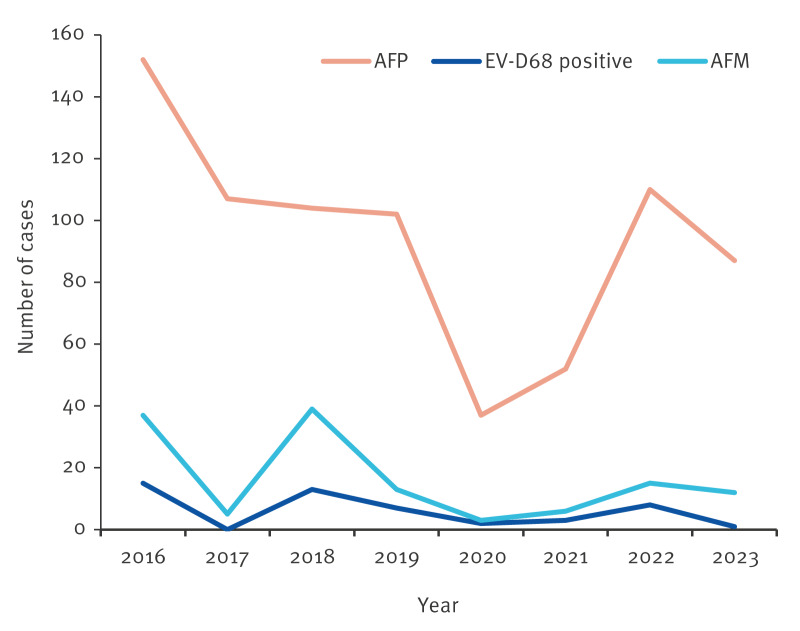
Annual totals of AFM (n = 130)^a^ or AFP (n = 750)^b^ case numbers obtained through a survey of several countries, Europe, 2016–2023

AFM: acute flaccid myelitis; AFP: acute flaccid paralysis; EV-D68: enterovirus D68.

^a^ Data on the respective AFM case numbers of the 15 countries in Europe providing this information are shown in the Table.

^b^ Data on AFP case numbers were obtained from four countries, including Norway (n = 84), Poland (n = 304), Spain (n = 277) and Switzerland (n = 85).

The total numbers of cases of AFP and AFM respectively found each year through the survey, as well as the annual numbers of AFM cases with EV-D68 are plotted from 2016 to 2023 in the Figure. While AFP and AFM annual case numbers seem to both present a peak in 2016 and minimum in 2020, as well as an increase in 2022 and decrease in 2023, there seems to be no concordance in some times (e.g. 2018). In addition to 2016, the annual AFM case numbers seem to peak in 2018 and 2022, with slight peaks also observable in those years for the numbers of EV-D68-positive AFM cases.

## Discussion

This survey conducted among clinicians and virologists, found 130 cases of AFM for 15 European countries between 2016 and 2023. While surveillance of AFP was reported to be active in eight European countries, our study found that structured surveillance for AFM was only performed in Norway.

While the study provides some indication of current AFM case numbers in Europe, these numbers should be interpreted with caution, not only because of the lack of structured AFM surveillance in most European countries, but also due to the selection of cases that are expected to be reported through a survey, i.e. likely more severe cases. Nevertheless, annual peaks in numbers of AFM cases apparent through the current investigation in 2016 and 2018 coincide with peaks noted in the same years in the United States (US), where systematic clinical surveillance is conducted by the Centers for Disease Control and Prevention (CDC) [4,9,15]. Also, it has been reported that EV-D68 circulation can be associated with the occurrence of AFM cases [2,4,7]. Within the US and Europe an upsurge of EV-D68 case numbers was seen in 2022 [16,17], but without a clear rise in AFM case numbers in the US. Our results, on the other hand, may be suggestive of a slight increase in AFM case numbers in Europe during that year.

Among the AFM cases found in the study, 37% were EV-D68-laboratory-confirmed. Moreover, 70% occurred in years 2016, 2018 and 2022, when EV-D68 circulation was more intense [16-18]. This finding may indicate that higher numbers of cases arise in times of elevated circulation of EV-D68. Alternatively, this result may arise from greater awareness or better reporting of AFM during years when EV-D68 is circulating.

The evolution in the yearly number of AFP cases did not appear always congruent with that of reported AFM cases from our survey. Due to AFP being only reported by four countries, the relevance of this observation is difficult to appraise. However, it may support that AFP surveillance without specification of the cause is not effective to keep track of the incidence of AFM. As the most common diagnosis in children with AFP is Guillain–Barré syndrome (GBS), variations in the yearly number of AFP cases might be mostly related to the number of patients with GBS [19].

Our report has several limitations. One of these stems from the lack of structured surveillance for AFM in Europe. Another is that the respondents to the questionnaire had heterogeneous backgrounds with variable connections to national surveillance structures, limiting the accuracy and completeness of numbers provided. Furthermore, data were not acquired for all countries and answers to survey questions were frequently incomplete, relying in some instances on personal awareness. It should also be considered that the numbers of AFM cases per country were based on clinical diagnoses, without information on the diagnostic work-up performed, which may have led to their over- or underestimations. We have focused on the association of AFM with EV-D68, but other viruses may be associated with AFM.

To gain better insight into the burden of AFM in Europe and establish connections between its upsurges and the circulation of associated viruses, it is crucial to closely monitor AFM incidence and to study the required samples from suspected cases. Tracking this would help to assess AFM as a health priority. Additionally, identifying and alerting on AFM upsurges within networks of clinicians, microbiologists, and public health specialists would raise awareness and the level of preparedness, ultimately improving the response to potential outbreaks.

To enhance our understanding on the incidence of AFM, we are setting up a European AFM repository. Cases will be included through the established network of European clinicians, incorporated in the ENPEN surveillance. Currently, 17 countries are represented in this network, and we aim to expand it for better coverage. To ensure consistent and accurate data collection, standardised case definition and guidelines for sampling and further testing will be provided. The current case definition for the future repository entails any case of suspected AFM, which will be further specified based on the diagnostic criteria [20]. By facilitating discussions of suspected cases within the established network and effectively communicating any upsurges of AFM cases or the circulation of associated EVs, we hope to improve both awareness and recognition of AFM across Europe.

## Data Availability

The data from this report can be made available upon request.

## Supplementary Data

497000 Supplementary Figure
