# Supplementary material for: Acute flaccid myelitis in Europe between 2016 and 2023: indicating the need for better registration
Source: Euro Surveill. 2025 May 29;30(21):2400579. doi: 10.2807/1560-7917.ES.2025.30.21.2400579 (PMC12124111; doi:10.2807/1560-7917.ES.2025.30.21.2400579)
Supplement: Supplementary Figure [file 2400579_SupplementaryFigure.pdf]

"This supplementary material is hosted by Eurosurveillance as supporting information alongside the article Acute flaccid myelitis in Europe 2016-2023: indicating the need for better registration on behalf of the authors, who remain responsible for the accuracy and appropriateness of the content. The same standards for ethics, copyright, attributions and permissions as for the article apply. Supplements are not edited by Eurosurveillance and the journal is not responsible for the maintenance of any links or email addresses provided therein."

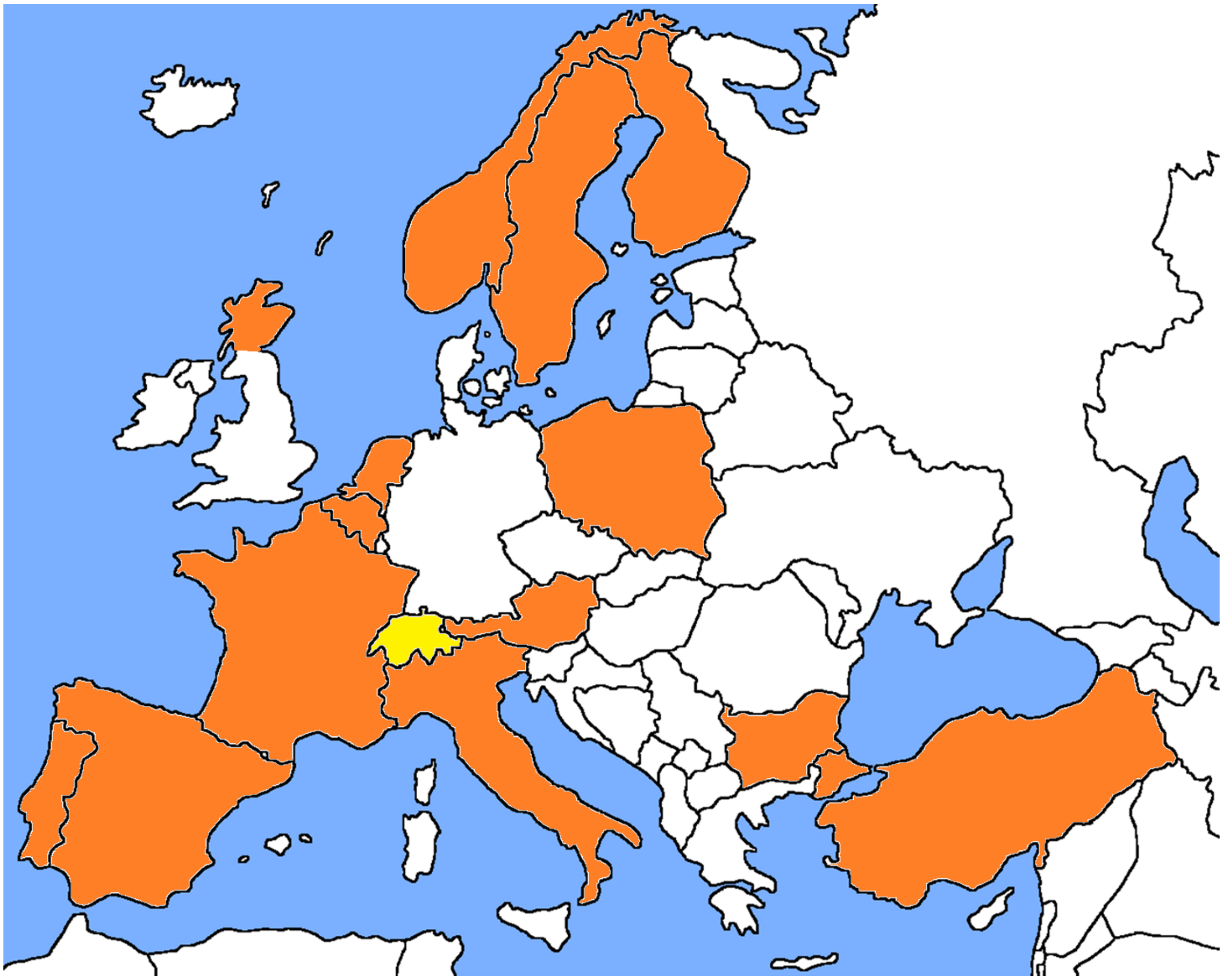

Supplementary figure: Map of Europe showing the countries from which a response was obtained for the current study, Europe, 2016-2023. From the orange-colored countries, information on the number of acute flaccid myelitis (AFM) cases was obtained. From the yellow country (Switzerland), only information on the number of acute flaccid paralysis (AFP) cases was available. From Spain, Norway and Poland (2022-2023), both information on the number of AFP and AFM cases was collected.
